# Folate as a Key Regulator of Animal Intestinal Homeostasis: From Metabolism to Microbiota and Barrier Function

**DOI:** 10.3390/ani16111744

**Published:** 2026-06-05

**Authors:** Yi Zheng, Yecheng Xu, Xin Wen, Xi Qiao, Tianzhao Yao, Linlin Wei, Huahua Du

**Affiliations:** Zhejiang Key Laboratory of Nutrition and Breeding for High-Quality Animal Products, College of Animal Sciences, Zhejiang University, Hangzhou 310058, China

**Keywords:** animal health, animal nutrition, animal production, folic acid, gut microbiota, mucosal immunity, one-carbon metabolism

## Abstract

Folate is a central nutrient in one-carbon metabolism, contributing to nucleotide biosynthesis, methionine cycling, methyl-donor supply, and epigenetic regulation. In animals, the intestine is both a principal site of folate absorption and a key target organ for folate action. This narrative review focuses primarily on livestock, poultry, aquaculture species, ruminants, and animal-source food enrichment, while also using rodent, human, and in vitro studies as mechanistic or translational evidence. We synthesize evidence on folate absorption, transport, and metabolism and evaluate the mechanisms through which folate influences intestinal health. Available evidence suggests that adequate folate supply may support epithelial renewal, tight-junction integrity, mucosal immune balance, antioxidant capacity, gut microbiota stability, short-chain fatty acid production, and epigenetic regulation of intestinal development. These effects have been reported in poultry, pigs, fish, ruminants, rodents, and maternal–offspring models. However, the evidence is uneven across species, and dose–response relationships, folate forms, bioavailability, and species-specific requirements remain major limitations for translating current knowledge into animal production. Future studies should compare folic acid, 5-methyltetrahydrofolate, natural reduced folates, microbiota-derived folate, and folate-producing probiotics; quantify the contribution of microbiota-derived folate to host methyl-donor pools; and develop precision strategies that integrate folate with other one-carbon nutrients, probiotics, and product-enrichment technologies.

## Simple Summary

Folate is a B vitamin required for normal growth, reproduction, and tissue renewal in animals. The intestine is a major site of folate absorption, but it is also an important biological target of folate supply. This review explains how folate from feed ingredients, supplements, and gut microbes may support intestinal development, strengthen barrier integrity, help maintain a balanced microbial community, and moderate excessive inflammation. Evidence from poultry, pigs, fish, ruminants, rodents, and maternal–offspring models suggests that folate nutrition may be useful not only for preventing deficiency but also for improving resilience during weaning, high production, metabolic stress, gestation, lactation, and early-life programming. However, the evidence remains uneven across species, and several mechanistic conclusions still rely on rodent, fish, human, or in vitro models rather than direct livestock studies. Practical strategies include optimizing the dose and chemical form of folate, combining folate with other one-carbon nutrients, applying folate-producing probiotics, and developing folate-enriched animal products. These approaches may help improve animal health, production efficiency, and the nutritional value of animal-derived foods.

## 1. Introduction

Folate, an essential B vitamin, serves as a primary carrier of one-carbon units and has long been recognized for its classical roles in preventing megaloblastic anemia, supporting fetal neural tube development, and regulating homocysteine metabolism [1,2,3]. However, recent advances in nutritional biochemistry, intestinal microbiology, and developmental biology have increasingly positioned the intestine as a key site of folate action. The intestinal epithelium undergoes rapid renewal, sustains continuous immune activity, and remains constantly exposed to dietary components and dense microbial communities. Consequently, the gut has a high demand for nucleotide synthesis, methyl-donor availability, and overall metabolic homeostasis. Folate integrates these processes through a network involving DNA synthesis, methionine cycling, S-adenosylmethionine (SAM) generation, and methylation reactions [4,5,6]. After uptake by intestinal transport systems such as the proton-coupled folate transporter (PCFT) and the reduced folate carrier (RFC), folate supports epithelial cell proliferation, differentiation, and repair [7]. Simultaneously, the gut microbiota can synthesize, consume, and compete for folate, indicating that luminal folate functions both as a host nutrient and a microbial metabolic resource [8,9]. Animal studies have extended the biological significance of folate from merely preventing deficiency to actively regulating intestinal barrier integrity, microbial metabolism, inflammatory responses, gut–liver axis homeostasis, maternal programming, and the development of functional animal products. Evidence from laying hens [10], breeder geese [11], goslings [12], weaned piglets [13], and grass carp [14] demonstrates that folate availability influences nutrient transport, villus morphology, tight-junction expression, inflammatory status, antioxidant capacity, short-chain fatty acid (SCFA) production, and microbial composition. Moreover, maternal folate status shapes offspring intestinal development and can exert lasting effects on gut function [15,16].

Although folate has been extensively reviewed in relation to human folate biology, neural tube defect prevention, and one-carbon metabolism [1,3,4], its role in animal intestinal homeostasis has received less integrated attention. In particular, the connections among folate absorption, microbial folate metabolism, epithelial barrier function, mucosal immunity, developmental programming, and production-stage-specific nutritional strategies have not been systematically discussed from an animal nutrition perspective. This gap is important because available animal studies suggest that folate status is associated with intestinal absorption, microbiota composition, barrier-related markers, and epithelial function in poultry, pigs, and fish [10,13,14].

Therefore, this review evaluates folate through four connected axes: host absorption and systemic distribution, microbial folate metabolism, epithelial-immune regulation, and production-stage-specific nutritional strategies. By integrating direct evidence from livestock, poultry, aquaculture species, and ruminants with mechanistic evidence from rodent, human, and in vitro models, this review aims to clarify how folate contributes to animal intestinal homeostasis and how folate-based strategies may be applied to improve animal health, productive performance, reproductive outcomes, and functional animal-source food enrichment.

## 2. Review Methodology

This article was prepared as a narrative review rather than a systematic review or meta-analysis. The literature search focused on folate, folic acid, 5-methyltetrahydrofolate (5-MTHF), one-carbon metabolism, and intestinal health in animal and experimental models. Searches were performed in PubMed, Web of Science, Scopus, Google Scholar, and the China National Knowledge Infrastructure using combinations of the following terms: “folate” OR “folic acid” OR “5-MTHF”; “intestinal absorption” OR “folate transporter” OR “PCFT” OR “RFC”; “gut microbiota” OR “short-chain fatty acids”; “intestinal barrier” OR “tight junction” OR “mucosal immunity”; “inflammation” OR “colitis”; “maternal folate” OR “developmental programming”; and “livestock”, “poultry”, “piglet”, “fish”, “laying hen”, “goose”, “ruminant”, “dairy cow”, “beef cattle”, “heifer”, “ewe”, “sheep”, “goat”, “rat”, or “mouse”. These searches initially identified approximately 140 potentially relevant records. After screening titles, abstracts, and available full texts for relevance to folate-related animal nutrition, intestinal health, microbiota, barrier function, maternal programming, and production outcomes, 69 references were included in the final narrative synthesis.

Priority was given to peer-reviewed studies that provided mechanistic information or animal data on folate nutrition, microbial folate production, intestinal morphology, barrier function, immune and inflammatory indices, maternal–offspring programming, or functional animal-product enrichment. Reviews and meta-analyses were used to contextualize mechanisms and evidence gaps. Chinese-language studies were included when they provided relevant animal data, and full texts were reviewed when available. where available, English titles or abstracts were used for reference formatting. Key terms were standardized before synthesis: “folate” was used as the umbrella term for biologically related folate vitamers; “folic acid” referred specifically to the oxidized synthetic monoglutamate form; “5-MTHF” referred to 5-methyltetrahydrofolate; “natural reduced folates” referred to reduced food/feed folates that commonly occur as polyglutamates; and “microbiota-derived folate” referred to folates synthesized by intestinal microorganisms. All figures were prepared as original schematic illustrations by the authors using BioRender.com. The figures were designed to synthesize mechanisms from the cited literature, and no previously published figures were reproduced. Articles were excluded when folate was not a central variable, when intestinal outcomes could not be interpreted, or when the study was not relevant to animal nutrition or gut health. Because this review is narrative in scope, no pooled effect estimates were calculated and no protocol was registered.

## 3. Folate Metabolism and Intestinal Homeostasis: From Absorption to Microbial Crosstalk

### 3.1. Folate Chemistry and One-Carbon Metabolism

Folate consists of a pteridine ring, *p*-aminobenzoic acid, and one or more glutamate residues. It serves as a coenzyme in one-carbon transfer reactions involved in purine synthesis, thymidylate synthesis, methionine cycling, and methylation reactions [3,4]. Natural feed folates usually occur as reduced polyglutamates, whereas fortified diets and feed additives commonly contain oxidized folic acid in monoglutamate form. These chemical forms differ in stability, deconjugation requirements, absorption efficiency, and metabolic fate; therefore, dietary intake does not necessarily reflect biologically available folate [1,5].

One-carbon metabolism determines not only nucleic acid synthesis but also methyl-donor availability and epigenetic status. Folate-mediated one-carbon metabolism can influence DNA methylation and thereby affect development and disease susceptibility [3]. More broadly, DNA and RNA methylation provide mechanistic links between nutritional status, developmental programming, and long-term phenotypes [4]. Because intestinal epithelial cells, mucosal immune cells, and embryonic tissues undergo rapid renewal and differentiation, they are particularly sensitive to folate availability and one-carbon metabolic flux. The stability, metabolic characteristics, advantages, limitations, and feed-use relevance of major folate forms and folate-oriented microbial strategies are compared in Table 1.

### 3.2. Intestinal Folate Absorption and Systemic Distribution

Intestinal folate utilization begins with luminal deconjugation and subsequent transmembrane transport, a process critically dependent on the PCFT. Defects in this transporter lead to hereditary folate malabsorption [17], and subsequent studies have further clarified the physiological regulation, structural biology, and disease relevance of PCFT and related transport systems [2,6,7]. Collectively, these findings indicate that folate absorption should be viewed as an integrated process involving small-intestinal uptake, hepatic conversion, and tissue redistribution, rather than one confined to the proximal intestine [18].

Human studies have shown that folate delivered to the colon can still be absorbed [19], and that folate synthesized by bacteria in the upper small intestine can be assimilated by the host [20]. These findings broaden the classical view of folate absorption and support the concept that colonic recovery and microbiota-derived folate may contribute to host folate homeostasis. However, they are interpreted here as translational evidence rather than direct livestock-production evidence. In poultry, everted intestinal sac studies demonstrate folate transport activity along multiple intestinal segments in laying hens, with stronger absorption in the duodenum and jejunum and residual capacity in the cecum [21,22].

**Figure 1:**
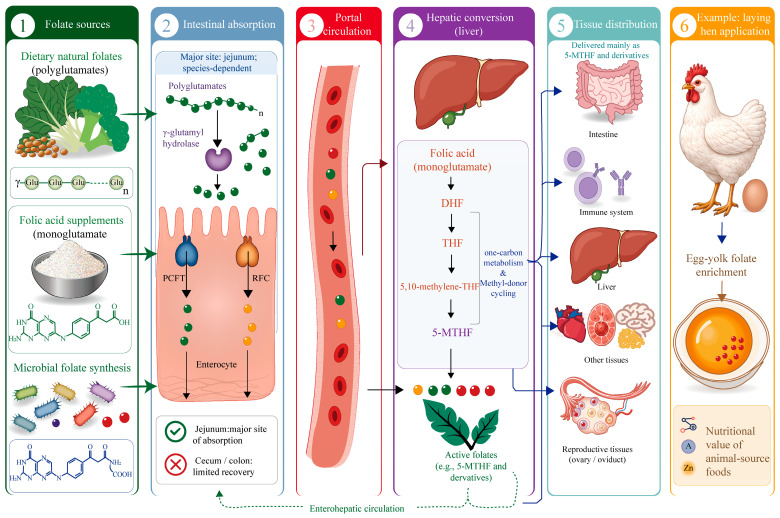
Overview of folate sources, intestinal absorption, hepatic conversion, and tissue distribution in animals. Dietary natural folates, supplemental folic acid, and microbiota-derived folates contribute to the host folate pool. After luminal deconjugation, folates are absorbed mainly in the proximal small intestine through transport systems such as PCFT and RFC, enter portal circulation, undergo hepatic conversion within one-carbon metabolism, and are redistributed to peripheral tissues and reproductive organs. In laying hens, redistributed folate can contribute to egg-yolk folate enrichment. Abbreviations: PCFT, proton-coupled folate transporter; RFC, reduced folate carrier; DHF, dihydrofolate; THF, tetrahydrofolate; 5-MTHF, 5-methyltetrahydrofolate. This figure was prepared as original schematic illustrations by the authors using BioRender.com.

Tissue distribution after absorption is influenced by species and production stage. In laying hens, folate absorbed by the intestine is transformed in the liver and redistributed to reproductive tissues and egg yolk [10]. Dietary supplementation increases egg folate content and improves the folate status of hens [23]. Folate-enriched eggs contain natural folate forms that remain relatively stable during common storage and cooking conditions [24,25]. Maternal supplementation can also affect folate status in eggs and offspring plasma and alter transporter expression in reproductive tissues [26]. These observations highlight both maternal effects and the practical value of folate for product enrichment. Figure 1 summarizes the major sources of folate, intestinal absorption routes, hepatic conversion, and tissue distribution in animals.

### 3.3. Microbiota-Derived Folate and Host Utilization

The involvement of gut microbes in folate homeostasis has shifted folate research from a classical nutrient-supply question to a host–microbe interaction framework. Microbial communities possess broad genetic potential for folate biosynthesis and transport [9]. B vitamins in the gut are used by both host tissues and commensal bacteria and participate in microbial competition and community assembly [27,28]. Thus, luminal folate availability is determined not only by dietary intake but also by microbial synthesis, uptake, and competition.

Research on microbiota-derived folate has progressed from identifying folate-producing organisms to evaluating host-level effects. Lactic acid bacteria and bifidobacteria can synthesize folate in a strain-dependent manner [29]. Administration of folate-producing bifidobacteria improves folate status in rats [30], whereas folate-producing lactic acid bacteria and folate-biofortified fermented products can influence microbial structure, SCFA generation, and inflammatory status [31,32].

The chemical form of folate may also shape microbial outcomes. In vitro fermentation studies comparing 5-MTHF with oxidized folic acid suggest that folate form, delivery site, and recipient microbial background can alter community composition and SCFA output [33]. Future discussions of “folate supplementation” should therefore integrate exogenous folate form, microbial utilization, colonic recovery, and host absorption as a connected system. Importantly, microbial folate production should not be equated directly with host folate utilization. At least three levels should be distinguished: microbial biosynthetic capacity, luminal folate availability, and host absorption followed by incorporation into systemic one-carbon metabolism. A bacterial strain or microbial community may increase folate production in the intestinal lumen, but the quantitative contribution of this pool to circulating 5-MTHF, hepatic methyl-donor metabolism, SAM availability, and tissue folate status depends on intestinal site, folate form, deconjugation, transporter expression, microbial competition, and host physiological state. Therefore, future animal studies should combine microbiome analysis with direct measurements of luminal folate, blood folate forms, tissue folate status, and one-carbon metabolites.

## 4. Folate and Intestinal Homeostasis: From Epithelial Renewal to Immune-Microbial Regulation

**Figure 2:**
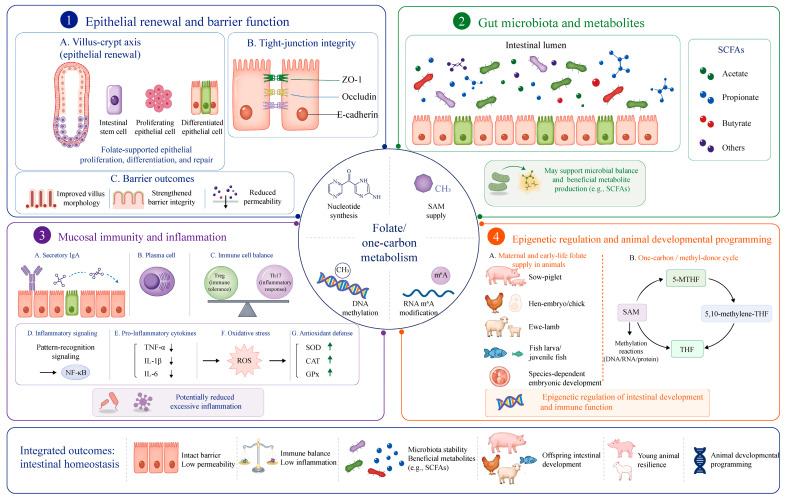
Major animal-oriented pathways by which folate regulates intestinal health. Folate-mediated one-carbon metabolism supports nucleotide synthesis and S-adenosylmethionine (SAM) supply, thereby contributing to epithelial renewal, tight-junction integrity, mucosal immune balance, antioxidant defense, microbiota–metabolite interactions, and epigenetic regulation. These convergent processes may help maintain intestinal homeostasis by reducing permeability, moderating excessive inflammation, supporting beneficial metabolites, and promoting developmental programming in embryos and young animals. Abbreviations: 5-MTHF, 5-methyltetrahydrofolate; THF, tetrahydrofolate; SAM, S-adenosylmethionine; SCFAs, short-chain fatty acids; IgA, immunoglobulin A; Treg, regulatory T cell; Th17, T helper 17 cell; ZO-1, zonula occludens-1; TNF-α, tumor necrosis factor alpha; IL, interleukin; NF-κB, nuclear factor kappa B; MLCK, myosin light-chain kinase; ROS, reactive oxygen species; SOD, superoxide dismutase; CAT, catalase; GPx, glutathione peroxidase; m6A, N6-methyladenosine. This figure was prepared as original schematic illustrations by the authors using BioRender.com.

The regulatory effects of folate on the intestine are multifaceted, arising from the coordinated interplay among epithelial renewal, barrier function, microbial metabolism, mucosal immunity, and epigenetic regulation. These pathways are presented as an integrated network rather than isolated mechanisms in Figure 2.

### 4.1. Epithelial Renewal and Barrier Function

The intestinal barrier comprises mechanical, chemical, immune, and microbial components. Epithelial integrity and mucosal barrier stability are essential for gut health. Because the epithelium renews rapidly, intestinal stem cells and differentiating epithelial cells require adequate nucleotide synthesis and methyl-donor availability. Folate supports DNA synthesis, SAM generation, and methylation reactions, thereby providing metabolic substrates for epithelial proliferation, differentiation, and repair [16,34].

Animal studies indicate that adequate folate supply improves villus morphology, villus height or villus-to-crypt ratio, and tight-junction-related gene expression [11,14]. In a colitis model, folate restored tight-junction proteins, including ZO-1, occludin, and E-cadherin, and inhibited abnormal activation of the PI3K/AKT/NF-κB/MLCK axis, thereby reducing intestinal permeability and inflammatory amplification [34]. These findings suggest that folate regulates the intestinal barrier through coordinated effects on morphology, epithelial renewal, junctional reconstruction, and signaling-pathway modulation.

### 4.2. Gut Microbiota and Microbial Metabolites

A second important pathway involves regulation of the intestinal physicochemical environment, microbial structure, and microbial metabolites. Rather than merely altering microbial abundance, folate may connect luminal conditions, microbial colonization, and fermentation outputs in ways that influence host physiology.

In weaned piglets, dietary folate level altered gastric and cecal pH, SCFA concentrations such as acetate and valerate, and Lactobacillus community structure [13]. In goslings, laying hens, and in vitro fermentation systems, folate alone or in combination with vitamin B12 altered cecal microbial diversity, dominant phyla, and SCFA output [10,12,32]. SCFAs serve as energy substrates for colonocytes and participate in the regulation of tight junctions, regulatory T-cell differentiation, inflammatory cytokine release, and gut–liver axis signaling. Accordingly, the microbiota-related significance of folate lies not only in compositional shifts but also in microbial metabolic outputs that affect barrier function, immunity, and systemic metabolism. It should be noted, however, that many animal studies report parallel changes in folate status, microbial composition, SCFA concentrations, barrier-related markers, and inflammatory indices, but these observations do not necessarily establish causality. Unless microbial transfer, pathway inhibition, gnotobiotic models, or targeted metabolite-rescue experiments are performed, such findings should be interpreted as associations or integrated response patterns rather than definitive evidence that microbiota-derived SCFAs directly mediate all folate effects. Therefore, the folate-microbiota-SCFA-barrier-inflammation axis should be considered a working mechanistic framework that requires further causal validation in production animals.

### 4.3. Mucosal Immunity and Inflammatory Responses

The mucosal immune system is continuously exposed to dietary antigens, microbial products, and commensal signals. It must balance immune tolerance with inflammatory defense. Folate can indirectly shape this environment by supporting epithelial renewal and one-carbon metabolism. It may also modulate signaling pathways such as NF-κB, MLCK, and p38, which regulate inflammatory cytokine release and oxidative stress [14,34].

In laying hens, breeder geese, and grass carp, folate supplementation has been associated with improved immune indices, enhanced barrier- and antioxidant-related factors, and moderated inflammatory responses [10,11,12,13,14]. Under stress or inflammatory conditions, folate can protect the mucosa by reducing inflammatory cascades, improving barrier integrity, and alleviating microbial dysbiosis [34,35]. Overall, folate appears to support mucosal immune homeostasis by maintaining epithelial structure, limiting excessive inflammation, and strengthening antioxidant defenses.

### 4.4. Epigenetic Regulation and Developmental Programming

Because folate lies at the center of one-carbon metabolism, its effects extend beyond epithelial proliferation and barrier repair. By controlling SAM supply, DNA methylation, and RNA N6-methyladenosine (m6A) modification, folate may participate in developmental programming of the intestine. Maternal nutrition, embryonic methyl-donor supply, and early microbial colonization jointly influence offspring gut development.

In a porcine model, maternal folic acid supplementation during gestation and lactation improved offspring body weight, villus development, nutrient-transporter expression, and intestinal stem-cell proliferation and differentiation [16]. Methyl-donor micronutrient supplementation also affected offspring microbial colonization, SCFA output, and metabolic phenotypes [15]. Conversely, maternal folate deficiency or an abnormal methyl-donor environment impaired offspring mucosal barrier development and intestinal maturation [36,37]. The long-term significance of folate for gut health should therefore be interpreted along a continuum linking maternal supply, intestinal development, epigenetic regulation, and adult health outcomes.

## 5. Folate and Animal Intestinal Health in Physiological and Pathological Contexts

**Figure 3:**
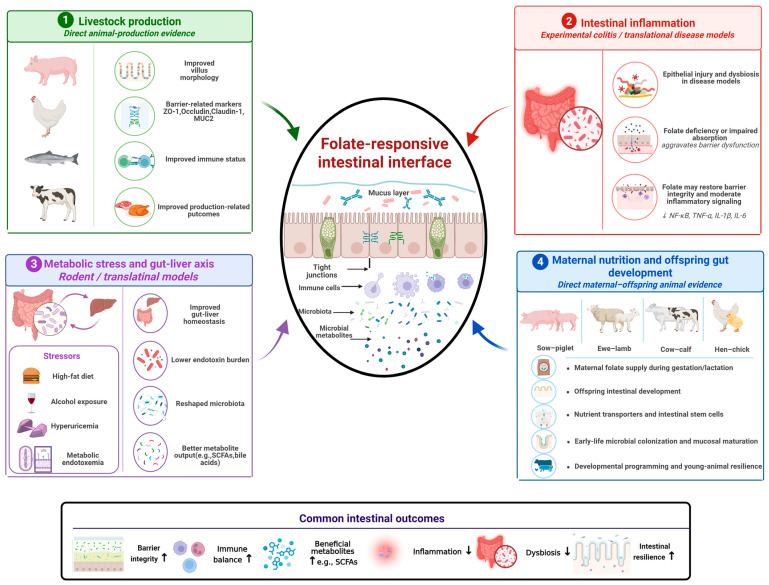
Folate-responsive intestinal outcomes in animals and translational models. The figure summarizes folate-associated responses across livestock production, intestinal inflammation, metabolic stress and gut–liver axis dysfunction, and maternal–offspring gut development. Direct animal evidence is separated from mechanistic or translational evidence derived from disease and metabolic-stress models. Common outcomes include improved barrier integrity, immune balance, microbial metabolites, reduced inflammation and dysbiosis, and enhanced intestinal resilience. Abbreviations: SCFAs, short-chain fatty acids; ZO-1, zonula occludens-1; MUC2, mucin 2; NF-κB, nuclear factor kappa B; TNF-α, tumor necrosis factor alpha; IL, interleukin. This figure was prepared as original schematic illustrations by the authors using BioRender.com.

The intestinal actions of folate extend beyond fundamental metabolic processes, exhibiting multifaceted physiological and pathological relevance in diverse contexts, including livestock production, intestinal inflammation, metabolic stress, and maternal nutrition. Accordingly, this section summarizes the roles of folate with respect to the following aspects: intestinal health and nutritional effects in livestock production, intestinal inflammation and mucosal injury, metabolic stress and gut–liver axis function, as well as maternal nutrition and offspring intestinal development (Figure 3).

### 5.1. Gut Health and Nutritional Effects in Livestock

In livestock, folate research initially focused on supplementation level, tissue enrichment, reproductive performance, and product nutritional quality. As knowledge of intestinal nutrition has deepened, evidence increasingly indicates that the production effects of folate are closely linked to intestinal absorption capacity, barrier stability, and microbial homeostasis. In production animals, folate-mediated one-carbon metabolism, epithelial renewal, immune regulation, and microbial metabolism may translate into improved growth performance, reproductive efficiency, stress resistance, and product quality.

Poultry models provide a relatively complete evidence chain. Folate absorbed in the intestine of laying hens participates in hepatic transformation and redistribution to the ovary and egg yolk [10,21]. Supplementation with folate alone or in combination with vitamin B12 can improve intestinal morphology, immune status, and cecal microbial structure in laying hens, breeder geese, and goslings [11,12]. These observations indicate that, under high egg production and reproductive demand, folate is valuable not only for meeting vitamin requirements but also for maintaining intestinal homeostasis as a foundation for productive and reproductive function.

Practical value is also evident in functional animal-product development. Dietary folic acid supplementation increases egg folate content, and enriched folate forms in egg yolk show favorable stability during storage and cooking [23,24,25]. In pigs, rabbits, and other mammals, folate effects are often linked to weaning, pregnancy, lactation, and high reproductive load. In weaned piglets, dietary folate level affected the gastrointestinal environment, microbiota, SCFA concentration, and organ development [13]. In rabbit does, folate supplementation interacted with litter size and reproductive load to influence maternal microbiota and reproductive performance [38].

Ruminants also represent an important application area for folate nutrition. Previous reviews and supplementation studies have investigated folic acid and related one-carbon nutrients in dairy cows, beef cattle, heifers, ewes, and maternal–fetal models, mainly in relation to lactation, reproduction, vitamin B12-dependent methyl metabolism, methionine cycling, and maternal–fetal nutrient supply [39,40,41,42,43]. In dairy cows, folic acid has often been studied together with vitamin B12 because folate-dependent one-carbon metabolism is closely linked to vitamin B12-dependent methionine synthase activity and methyl-donor balance [39,40,41,42]. In sheep, maternal periconceptional B-vitamin and methionine supply has also been used as a model for one-carbon-related developmental programming [43]. In addition, one-carbon metabolite supplementation in beef heifers and fetuses altered vitamin B12, folate, and methionine-cycle metabolites, supporting the relevance of folate-related methyl-donor metabolism in ruminant pregnancy [44]. However, these studies should be distinguished from direct intestinal-homeostasis evidence. Compared with poultry, pigs, and fish, fewer ruminant studies have directly tested whether folate supplementation regulates intestinal barrier integrity, hindgut microbiota, SCFA profiles, or mucosal immune markers. Thus, ruminants are an important but still underexplored species group for folate-mediated intestinal homeostasis.

Overall, folate effects in production animals can be considered at three levels: first, support of intestinal absorption and nutrient transport during rapid growth, egg production, or reproduction; second, maintenance of barrier function, local immunity, and microbial stability under weaning, high-production, and reproductive stress; and third, conversion of gut-health effects into productive benefits through egg folate enrichment, improved reproductive outcomes, and optimized offspring phenotypes. To clarify the species-specific evidence, representative animal studies on folate-related interventions and their intestinal or production-related outcomes are summarized in Table 2.

### 5.2. Intestinal Inflammation and Mucosal Injury

The relationship between folate and intestinal inflammation is first reflected in altered folate status. The evidence discussed in this subsection is mainly mechanistic or translational and should not be interpreted as direct animal-production evidence. A meta-analysis reported lower mean serum folate concentrations in patients with inflammatory bowel disease than in healthy controls, whereas changes in vitamin B12 were less consistent [45]. Classic clinical studies also showed that sulfasalazine can inhibit folate absorption and contribute to folate deficiency in ulcerative colitis [46,47]. Thus, in inflammatory bowel disease, folate insufficiency may be both a consequence of disease activity or impaired absorption and a factor that weakens mucosal repair.

Mechanistic studies suggest that folate may modulate disease-related processes. Folate attenuated experimental colitis by restoring tight-junction structure and inhibiting abnormal PI3K/AKT/NF-κB/MLCK activation [34]. In inflammatory bowel disease, folate may therefore be relevant not only for deficiency correction but also for limiting barrier damage, complications, and inflammatory progression [48]. Earlier work proposed that folate and butyrate represent complementary mechanisms in intestinal disease prevention and treatment: folate supports epithelial metabolism and methyl-donor cycling, whereas butyrate supports fermentation-derived energy supply and local microbial ecology.

Folate has also been investigated in the context of the transition from chronic inflammation to colorectal cancer risk. Emerging evidence indicates a complex, context-dependent relationship between folate status and colorectal carcinogenesis, which is modulated by factors such as dose, timing, and baseline host characteristics [49,50]. In animal models of ulcerative colitis-associated colorectal cancer, dietary folate supplementation has been shown to suppress the development of high-grade lesions [51]. Systematic reviews generally support an inverse association between dietary folate intake and colorectal cancer risk, although the magnitude and significance of this effect vary depending on study design, supplementation dose, and disease stage [52]. Collectively, these findings suggest that in the setting of inflammation and mucosal injury, folate may serve multiple protective roles—as an indicator of nutritional status, a factor supporting intestinal barrier function, and an epigenetic regulator.

### 5.3. Metabolic Stress and Gut–Liver Axis Function

Folate research has expanded from local intestinal protection to metabolic stress and gut–liver axis regulation. Under high-fat diet, alcohol exposure, postpartum weight retention, and hyperuricemia, folate may not replace a single metabolic pathway; rather, it may improve intestinal barrier function, reshape microbial metabolism, and reduce endotoxin burden, thereby indirectly alleviating hepatic lipid accumulation, inflammation, and metabolic imbalance.

In offspring of high-fat-diet-fed rat dams, maternal folic acid supplementation was associated with microbiota remodeling, reduced lipopolysaccharide exposure, enhanced tight-junction expression, and reduced hepatic steatosis [53]. In an alcohol-exposure model, folic acid improved gut–liver axis homeostasis through modulation of the intestinal microbiota [54]. Other studies in high-fat obesity, mitochondrial metabolism, and postpartum weight-retention models reported that folate supplementation was accompanied by lower body-weight gain, modified branched-chain amino acid metabolism, and reduced endoplasmic reticulum stress [55,56,57]. These findings suggest that folate can reshape the host metabolic environment through intestinal mechanisms.

Hyperuricemia studies further support this view. Folate intervention altered gut microbial composition in hyperuricemic rats [33]. Folic acid combined with zinc improved uric acid production and excretion and restored microbial metabolic functions in high-purine diet-induced hyperuricemia [58]. Under metabolic stress and gut–liver axis dysregulation, the core role of folate may be to maintain intestinal stability, limit inflammatory spillover, and optimize microbial metabolic output to restore systemic metabolic balance.

### 5.4. Maternal Nutrition and Offspring Intestinal Development

Maternal folate supply is a major theme in folate biology with long-term relevance for intestinal development. The key question is not whether folate simply promotes growth, but how methyl-donor supply, epigenetic modification, and maternal–fetal nutrient transfer program the trajectory of offspring gut development.

Maternal folic acid supplementation during gestation and lactation improved villus development, nutrient transport, and intestinal stem-cell proliferation and differentiation in offspring piglets [16]. Methyl-donor supplementation also altered offspring gut microbiota, SCFA output, and metabolic profiles [15]. In contrast, maternal folate deficiency or an abnormal methyl-donor environment impaired offspring intestinal mucosal barrier development and maturation [36,37]. Maternal–offspring transfer studies further indicate that maternal folate status affects embryonic and early-life folate supply and organ development [26,59].

Developmental disease studies, including Hirschsprung disease-related evidence, provide additional mechanistic perspectives but should not be interpreted as direct production-animal evidence. High-dose maternal folate supplementation has been explored in relation to colonic EDNRB expression and mechanisms of Hirschsprung disease [60], and folate regulation of m6A methylation has been linked to intestinal developmental abnormalities [61]. Studies of ulcerative colitis epigenetics and MTHFR polymorphisms also indicate connections among folate deficiency, abnormal gene methylation, and disease susceptibility [62]. Folate in maternal nutrition, intestinal developmental abnormalities, and disease risk should therefore be interpreted within a developmental-programming framework rather than as a simple deficiency model.

## 6. Nutritional Regulation Strategies and Application Prospects

**Figure 4:**
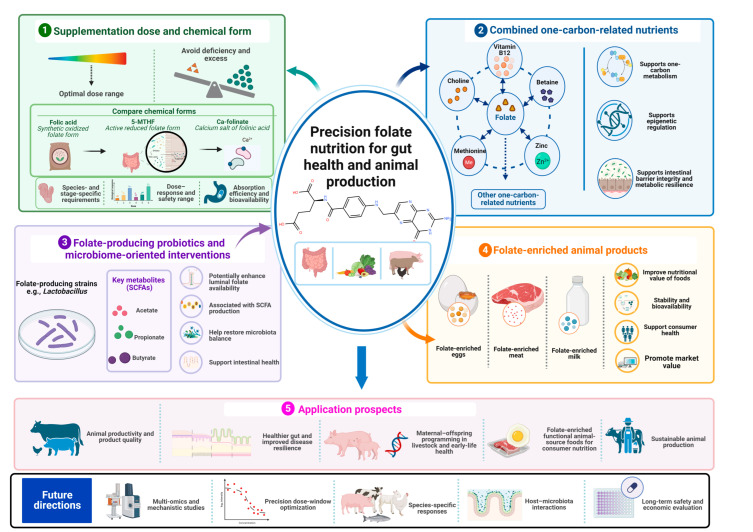
Nutritional regulation strategies and application prospects of folate for intestinal health. Proposed folate-based strategies include optimizing supplementation dose and chemical form, combining folate with one-carbon-related nutrients, applying folate-producing probiotics or microbiome-oriented interventions, and developing folate-enriched animal products. These approaches may support precision folate nutrition, gut health, animal productivity, product quality, maternal–offspring developmental programming, and sustainable animal production. Future work should integrate multi-omics, dose-window optimization, species specificity, and host–microbiota interactions. Abbreviations: 5-MTHF, 5-methyltetrahydrofolate; Ca-folinate, calcium folinate; B12, vitamin B12; Zn, zinc; CH3, methyl group; SCFAs, short-chain fatty acids; SAM, S-adenosylmethionine. This figure was prepared as original schematic illustrations by the authors using BioRender.com.

Based on current evidence, the application of folate in regulating animal gut health has advanced beyond the mere correction of deficiency toward more targeted strategies, including precision nutrition, microbial modulation, maternal programming, and the development of functional animal products. Figure 4 summarizes the primary intervention strategies and their anticipated outcomes.

### 6.1. Supplementation Level and Chemical Form

The first practical questions are how much folate should be supplied and which chemical form should be used. Animal studies indicate that appropriate folate levels can improve intestinal morphology, immune status, microbial structure, and product enrichment. However, very high supplementation does not necessarily produce linear benefits and may alter deposition of other nutrients or disturb metabolism [5,63]. Excessive folic acid may exceed the capacity for reduction and conversion to active folate forms, potentially increasing unmetabolized folic acid [64,65]. Moreover, folate functions within an interconnected methyl-donor network involving vitamin B12, methionine, choline, betaine, and SAM-dependent methylation reactions; therefore, imbalanced supplementation may disturb one-carbon metabolic homeostasis [3,4]. Folate supplementation should therefore be designed according to species, production stage, basal dietary folate, intestinal health status, target product, and safety boundaries.

Chemical form is another key determinant of efficacy. Natural folates are mostly reduced polyglutamates, whereas feed additives frequently use oxidized folic acid, and the dominant active forms in blood and tissues include 5-MTHF [1,5,66]. These forms differ in stability, deconjugation requirements, transport efficiency, and microbial effects. In vitro fermentation studies suggest that 5-MTHF and unreduced folic acid can have distinct effects on microbial composition and SCFA output [33]. Future studies should compare folic acid, 5-MTHF, and natural folate forms across species, production stages, and intestinal sites.

### 6.2. Combined Supplementation with Vitamin B12 and Other Methyl Donors

Folate acts within a network rather than in isolation. Vitamin B12, choline, betaine, methionine, and trace elements such as zinc can influence one-carbon metabolism, methyl-donor balance, antioxidant capacity, and microbial functions. Vitamin B12 is a key cofactor for methionine synthase; imbalances between folate and vitamin B12 can influence homocysteine remethylation, SAM generation, and methylation status. In production animals, combined supplementation of folate and vitamin B12 has a strong mechanistic basis. Poultry studies show that this combination can improve intestinal morphology, immune indices, and cecal microbiota in breeder geese and goslings [11,12].

Other nutrients may provide complementary benefits. Folic acid and zinc improved hyperuricemia through microbial and metabolic mechanisms [58]. In beef heifers and fetuses, one-carbon metabolite supplementation altered vitamin B12, folate, and methionine-cycle metabolites, supporting the relevance of methyl-donor balance in ruminant pregnancy [44]. Future strategies should therefore move from single-vitamin supplementation toward one-carbon nutrient combinations that integrate folate, vitamin B12, choline, betaine, methionine, and trace elements according to production stage and health objective.

### 6.3. Folate-Producing Probiotics and Microecological Regulation

Folate-producing probiotics represent a promising direction for intestinal folate regulation. Lactic acid bacteria and bifidobacteria differ markedly in their folate biosynthetic capacity [67,68]. Compared with direct folate supplementation, folate-producing strains may provide dual benefits: they may increase luminal folate availability and potentially contribute to host folate status, while also improving microbial ecology through competitive colonization, luminal acidification, SCFA generation, and suppression of pathogen expansion.

Functional studies show that folate-producing bifidobacteria can improve folate status in rats [30]. However, the use of folate-producing probiotics in livestock and poultry remains underexplored, and future studies should determine whether these strains can survive feed processing, colonize the gastrointestinal tract, produce folate in situ, and contribute measurably to host folate pools. Folate-producing lactic acid bacteria can also modulate beneficial taxa and promote butyrate production [31,32]. Future work should evaluate folate-producing probiotics not only for folate output in vitro but also for strain safety, folate-biosynthesis genes, feed-processing tolerance, gastrointestinal survival, intestinal-site-specific folate release, effects on resident microbiota, and measurable host utilization. Potential application scenarios include weaning stress in piglets, peak laying or reproductive stress in poultry, larval and juvenile stages in aquaculture, intestinal inflammation risk, and rumen or hindgut microbial modulation in ruminants. These strains may also be combined with conventional probiotics, prebiotics, or postbiotics as part of microbiome-oriented feeding strategies.

### 6.4. Development of Folate-Enriched Animal Products

Folate-enriched animal products have high translational potential. Laying hens are an important model: dietary folic acid increases yolk 5-MTHF content and, under appropriate conditions, can improve performance and egg quality [23,66]. Folate-enriched eggs retain natural folate forms during common storage and cooking, supporting their feasibility as functional foods [24,25]. Recent studies on combined regulation with folate, vitamin B12, and other nutrients suggest that synergistic strategies may further improve egg folate deposition.

Three issues require attention. First, folate enrichment can show dose–response plateaus; excessive supplementation may not increase enrichment efficiency and may affect fatty acid, amino acid, or other nutrient composition. Second, enriched products should be assessed for folate form, stability, bioavailability, and sensory quality, rather than total folate content alone. Third, animal health, product quality, and consumer nutritional needs should be considered together so that functional product development remains integrated with intestinal health management.

## 7. Conclusions and Future Directions

Folate connects one-carbon metabolism, epithelial renewal, mucosal immunity, gut microbiota, and epigenetic regulation. Current evidence indicates that folate functions in animals extend beyond traditional vitamin nutrition to include intestinal barrier maintenance, microbial metabolic regulation, inflammatory buffering, gut–liver axis homeostasis, and maternal nutritional programming. In livestock and poultry, these intestinal effects may translate into improved growth, reproduction, stress resistance, and product nutritional quality. In inflammation, metabolic stress, and offspring development, folate may provide barrier protection, microbial remodeling, and long-term regulation of health trajectories.

Several questions remain unresolved. First, the absorption efficiency, microbial utilization, and tissue distribution of different folate forms are not well characterized across animal species. Second, dose, timing, and species specificity require multilayer validation. Third, the quantitative contribution of microbiota-derived folate to host methyl-donor pools, and its interaction with exogenous folate, remain unclear. Fourth, synergistic mechanisms among folate, vitamin B12, choline, betaine, zinc, and other one-carbon nutrients need to be defined, including safe boundaries for precision nutrition.

Future research should shift from single-nutrient supplementation toward integrated regulation of nutrition, microbiota, barrier function, and epigenetics. Dose–response models should be established for different species and production stages, and multi-omics approaches should be used to identify key microbes, metabolites, and epigenetic biomarkers. Translational research on folate-enriched products, folate-producing probiotics, and one-carbon nutrient combinations may provide new strategies for improving animal production efficiency, preventing intestinal disorders, and developing functional animal-source foods. Practically, folate should be considered not only as a deficiency-prevention vitamin but also as a precision nutritional tool that can be optimized according to species, production stage, intestinal health status, basal diet, microbial context, and target product. When appropriate dose windows and folate forms are defined, folate-based strategies may contribute to healthier animals, improved production efficiency, enhanced reproductive outcomes, and higher-value animal-source foods.

## Figures and Tables

**Table 1:** Comparison of folate forms and folate-oriented microbial strategies in animal feeding.

Folate Form	Stability	Absorption and Metabolism	Advantages	Limitations	Relevance to Animal Feed
Folate	Less stable; sensitive to heat, oxidation, processing, and storage	Usually polyglutamates; requires deconjugation for absorption; enter one-carbon metabolism after conversion to active forms	Naturally present in feed ingredients; biologically relevant reduced forms	Variable content/bioavailability; processing degradation	Important folate source; hard to quantify in formulated diets
Folic acid	High chemical stability; suitable for premixes and feed processing	Synthetic oxidized monoglutamate; requires reduction and conversion before entering active folate pools	Cost-effective, stable, and widely used in supplementation and enrichment studies	High intake yields no extra benefit and risks folic acid buildup or metabolic disruption	Primary supplement for poultry, pigs, fish, and potentially ruminants
5-MTHF	Less stable than folic acid but a major circulating active folate form	Enter methyl-donor metabolism more directly; linked to methionine cycling and SAM generation	Potentially higher biological relevance for methylation-related outcomes	Costly; limited feed evidence; stability unproven	Promising for precision nutrition, reproduction, and developmental programming
Microbiota-derived folate	Depends on microbial strain, intestinal site, substrate availability, and microbial competition	Lumen-produced; utilization depends on site, form, and absorptive capacity	Links diet, microbiota, SCFA production, and host folate status	Microbial production does not necessarily equal absorption or systemic methyl-donor contribution	Relevant to gut health, but its quantitative contribution remains unclear
Folate-producing probiotics	Strain-specific; viability during feed processing, storage, and gastrointestinal transit	Produce folate in situ while affecting microbial ecology and fermentation	Dual potential: endogenous folate supply and probiotic effects	Requires strain screening, safety evaluation, gastrointestinal survival testing, and host-utilization validation	Potentially useful for weaning stress, reproductive animals, aquaculture juveniles, gut inflammation, and microbiota-targeted feeding

**Table 2:** Summary of animal studies evaluating folate-related interventions and intestinal or related outcomes.

Species	Reference	Model/Stage	Intervention	Dose and Duration	Key Outcomes	Key Conclusion
Laying hens	[10]	Folate absorption/cecal microbiota	Folic acid	0, 1, 6, and 24 mg/kg feed; 8 weeks	Serum folate plateaued at 6 mg/kg; microbiota and folate transport/conversion genes were altered.	6 mg/kg folic acid supported egg folate enrichment without obvious adverse effects.
Laying hens	[23]	Egg folate enrichment/strain comparison	Crystalline folic acid	0, 2, 4, 8, 16, 32, 64, and 128 mg/kg diet; 21 d	No intestinal endpoints; egg and plasma folate increased, homocysteine decreased, laying performance unchanged.	Folic acid enriched egg folate, with deposition largely saturated at ≥2 mg/kg diet.
Breeder geese	[11]	34-week-old laying Wulong breeder geese; 2 × 3 factorial dietary folic acid × vitamin B12 trial	Dietary folic acid + vitamin B12	Folic acid: 0.5 or 2.0 mg/kg diet; vitamin B12: 15, 25, or 75 μg/kg diet; 1-week pretrial followed by 18-week feeding trial	Jejunal morphology, serum immunoglobulins, and crude protein utilization responded to folic acid and vitamin B12.	Combined folic acid and vitamin B12 supported jejunal structure, immunity, and nutrient utilization.
Goslings	[12]	1-day-old Wulong goslings; cecal microbiota and growth performance	Folic acid + vitamin B12	Folic acid: 0.55 or 2.50 mg/kg diet; vitamin B12: 0.009, 0.018, or 0.036 mg/kg diet; 4 weeks	Folic acid plus vitamin B12 improved growth, microbial richness, and cecal microbial composition.	The combination may improve gosling growth partly by optimizing cecal microbiota.
Weaned piglets	[13]	Weaning/early growth	Dietary folic acid	0, 3, 9, and 18 mg/kg diet; 14 d	Folic acid altered pH, increased cecal acetate and valerate, and enriched Lactobacillus species.	Dietary folic acid improved gut conditions and microbial fermentation.
Grass carp	[14]	Subadult grass carp; intestinal epithelial function and barrier regulation	Folic acid	0.57, 1.11, 1.53, 2.08, 2.64, and 3.15 mg/kg diet; 8 weeks	Folic acid improved intestinal development, goblet cells, barrier markers, folate/methionine metabolism.	Folic acid strengthened intestinal epithelial function and barrier integrity.
Offspring piglets	[16]	Maternal folic acid supplementation during gestation and lactation; offspring at weaning	Maternal folic acid supplementation	Sows received basal diet or 15 mg/kg folic acid from gestation to lactation until piglet weaning at 35 d	Maternal folic acid improved offspring villus development, nutrient transporters, ZO-1, cytokine profile, and stem-cell markers.	Maternal folic acid promoted offspring intestinal development and epithelial renewal.
Rabbit does	[38]	Lactating-pregnant Hyplus rabbit does under a 49 d breeding model; different litter sizes	Folic acid	0, 15, 30, and 45 mg/kg diet during lactation-pregnancy breeding cycle	Folic acid affected kit growth, reproductive hormones, and fecal taxa related to fiber degradation and anti-inflammatory function.	Folic acid supported reproduction, kit growth, and microbial function, with dose depending on litter size.
Dairy cows	[40]	Multiparous Holstein cows during lactation; folate metabolism and methyl-donor balance	Dietary folic acid ± rumen-protected methionine	Folic acid: 0, 3, or 6 mg/kg BW per day; rumen-protected methionine: 0 or 18 g/day; 305 d lactation	No direct intestinal endpoints; folic acid increased serum/milk folate and affected one-carbon metabolites.	This supports folate–methionine–B12 interactions, but ruminant intestinal evidence remains limited.

## Data Availability

No new data were created or analyzed in this study. Data sharing is not applicable to this article.
